# Recognizing and Intervening on Elder Abuse, Neglect, and Exploitation in the Age of COVID-19

**DOI:** 10.1093/geroni/igab046.278

**Published:** 2021-12-17

**Authors:** Pi-Ju Liu, Pamela Teaster

## Abstract

The rapid outbreak of coronavirus disease 2019 (COVID-19) caused by the severe acute respiratory syndrome coronavirus 2 (SARS-CoV-2) has led to a global pandemic. Public health measures to prevent the spread of COVID-19, such as social distancing and self-quarantine, have drastically altered people’s lives and led to social isolation, financial instability, and disrupted access to healthcare and social services. Older adults have not only borne the brunt of the highest COVID-19 mortality rates, but recent studies also describe growing reports of elder mistreatment. It is necessary to attend to these age-related disparities during the remainder of the COVID-19 pandemic and future health crises. This symposium includes four presentations on researchers’ findings in elder mistreatment during the COVID-19 pandemic. Dr. E-Shien Chang will compare prevalence of elder mistreatment before and since the pandemic, and highlight associated risk and protective factors during the pandemic. Dr. Lena Makaroun will examine changes in elder mistreatment risk factors among caregivers during the pandemic. Dr. Pamela Teaster will present Adult Protective Services’ (APS) policy and practice changes in response to the pandemic to demonstrate the pandemic’s impact on service providers. Lastly, Dr. Pi-Ju (Marian) Liu will appraise elder mistreatment victims’ awareness of COVID-19 and their unmet needs during the pandemic. Following the four presentations, Dr. Pamela Teaster will moderate a discussion on how elder mistreatment is a growing concern, especially during the pandemic, and what service providers are doing to protect older adults.

